# Perioperative risk factors for osteoporosis after radical gastrectomy for gastric cancer

**DOI:** 10.1186/s12893-024-02717-4

**Published:** 2024-12-27

**Authors:** Hyun-Jae Lee, Hye Seong Ahn, Dong-Seok Han

**Affiliations:** 1 Department of Surgery, Seoul National University Hospital, Seoul, 03080 Korea; 2 Department of Surgery, Seoul Metropolitan Government-Seoul National University Boramae Medical Center, Boramae-ro 5-20, Dongjak-gu, Seoul, 07061 Republic of Korea

**Keywords:** Osteoporosis, Gastrectomy, Gastric cancer, Risk factor, Alkaline phosphatase, Calcium

## Abstract

**Background:**

Osteoporosis, a frequent complication of gastrectomy, increases with age, and the average age of gastric cancer patients continues to rise. This study aims to analyze perioperative factors of osteoporosis after radical gastrectomy.

**Materials and methods:**

This retrospective cohort study included patients who underwent dual-energy-X-ray absorptiometry after gastrectomy due to gastric cancer between 2016 and 2019 at Seoul Boramae Medical Center. Data were analyzed from before surgery to 12 months after surgery. Statistical analyses identified osteoporosis risk factors among perioperative factors.

**Results:**

Among 189 patients, osteoporosis was diagnosed in 72 patients and peaked at 36 months postoperatively (46.3%; 24 out of 54) with the lowest mean T score of -3.34 although Ca and vitamin D supplements were prescribed to 157 patients (83.1%) on average 32.4 months postoperatively. In multivariate analysis, age (*P* = 0.002; Adjusted OR: 1.059, 95% CI: 1.020–1.098), body weight (*P* = 0.009; Adjusted OR: 0.950, 95% CI: 0.914–0.987), sex (*P* = 0.021; Adjusted OR: 2.322, 95% CI: 1.138–4.739), and serum ALP (*P* = 0.009; Adjusted OR: 1.023, 95% CI: 1.006–1.040) were significant preoperatively. Additionally, age (*P* = 0.005; Adjusted OR: 1.067, 95% CI: 1.020–1.116), serum Ca (*P* = 0.046; Adjusted OR: 0.357, 95% CI: 0.130–0.980), Cr (*P* = 0.003; Adjusted OR: 0.021, 95% CI: 0.002–0.268), and ALP (*P* = 0.014; Adjusted OR: 1.017, 95% CI: 1.003–1.030) were observed significantly at 12 months postoperatively.

**Conclusions:**

38.1% of patients were diagnosed with osteoporosis after radical gastrectomy, despite Ca and vitamin D supplements. Age, body weight, sex, serum Ca, Cr, and ALP correlated with osteoporosis perioperatively.

**Supplementary Information:**

The online version contains supplementary material available at 10.1186/s12893-024-02717-4.

## Background

According to the National Cancer Information Center in Korea, the incidence of stomach cancer was 55.3 per 100,000 people in 2021. Despite a steady decline, it was still the fourth-highest incidence among Koreans [1]. Additionally, stomach cancer was the fifth most common cancer worldwide, with 1.1 million cases annually, and it accounted for the fourth most common cause of cancer-related deaths globally, with approximately 800,000 deaths [2, 3]. The number of elderly patients has increased in recent years. According to a national survey conducted by the Korean Gastric Cancer Association in 2021, the proportion of gastric cancer patients aged over 70 increased from 9.1% in 1995 to 28.8% in 2019 [4].

It is important for cancer patients to return to their daily lives after surgical treatment and to prevent complications such as weight loss, acid reflux, anemia, and brittle bones [5, 6]. Osteoporosis, a condition characterized by brittle bones, is one of the chronic complications in stomach cancer patients [7]. Due to the bypass of food and reduced absorption time after gastrectomy, the absorption of many nutrients, particularly calcium and vitamin D, can be impaired [8]. Lim JS et al. reported that the prevalence of osteoporosis in gastric adenocarcinoma patients aged over 50 years was 39.6%, with the osteoporosis rate of the lumbar spine being 29.8% in males and 54.5% in females [9]. Therefore, as the number of elderly gastric cancer patients increases, the number of gastric cancer patients with osteoporosis as a complication is also likely to rise.

Previous studies have reported age, sex, preoperative BMI, weight loss after surgery, and preoperative levels of alkaline phosphatase and creatinine as risk factors [10–12]. However, most of these risk factors are preoperative, and postoperative factors, such as weight change after surgery or blood chemistry tests, have not been adequately considered.

Furthermore, previous studies have shown that low socioeconomic status is associated with osteoporosis [13, 14], but no study has investigated the relationship between socioeconomic status and osteoporosis in patients who have undergone gastrectomy.

Thus, this study aims to identify the risk factors associated with the development of osteoporosis following gastrectomy, including postoperative factors and socioeconomic status, at Seoul Boramae Medical Center, a hospital that serves medically vulnerable populations. And this study investigates the risk factors associated solely with gastrectomy for gastric cancer, excluding factors that may contribute to secondary osteoporosis, such as Cushing’s syndrome, hyperparathyroidism, thyrotoxicosis, severe liver cirrhosis, renal failure requiring dialysis, rheumatoid arthritis, hematologic diseases, and patients on steroids or hormone replacement therapy [15–22].

## Materials and methods

### Study population

The study enrolled patients who underwent gastrectomy for gastric cancer, excluding those who had palliative surgery, from January 1, 2016, to December 31, 2019, at Seoul Boramae Medical Center, and who underwent dual-energy X-ray absorptiometry (DXA) at least once. The gastrectomy procedures were performed with regional lymphadenectomy (D1 plus or D2) according to the Korean guidelines for gastric cancer [23]. We excluded patients who underwent gastrectomy for other stomach-origin malignancies, such as gastrointestinal stromal tumor, leiomyosarcoma, gastric lymphoma, or gastric neuroendocrine tumor.

The exclusion criteria were as follows: patients who declined to undergo DXA or were lost to follow-up before receiving a DXA recommendation, patients who experienced relapse during the follow-up period, patients who underwent remnant gastrectomy, patients diagnosed with osteoporosis before gastrectomy, patients diagnosed with Cushing’s syndrome, hyperparathyroidism, thyrotoxicosis, severe liver cirrhosis (Child-Pugh grade ≥ B) [24], renal failure requiring peritoneal dialysis or hemodialysis, rheumatoid arthritis, or hematologic diseases (e.g. multiple myeloma, systemic mastocytosis, thalassemia, hemophilia, sickle cell disease, monoclonal gammopathy, and pernicious anemia), patients who underwent gastrectomy for other stomach-origin malignancies, such as gastrointestinal stromal tumor, leiomyosarcoma, gastric lymphoma, or gastric neuroendocrine tumor, and patients on steroids or hormone replacement therapy.

Calcium and vitamin D supplements were administered based on specific criteria. One tablet of Dicamax D^®^(Ca carbonate 263.15 mg/cholecalciferol 10 mg, Dalim BioTech, Republic of Korea) or CAL-D3 ^®^(Ca citrate hydrate 480 mg/cholecalciferol 10 mg, Iworld Pharm, Republic of Korea) was administered when serum Ca < 9 mg/dL or vitamin D < 20 ng/mL or osteopenia was identified. One tablet of Dicamax 1000^®^(Ca carbonate 1315.78 mg/cholecalciferol 10 mg, Dalim BioTech, Republic of Korea) or 2 tablets of Wondercal D^®^(Ca citrate 850 mg/cholecalciferol 5 mg, Chongkundang, Republic of Korea) was administered when serum Ca < 8.7 mg/dL or osteoporosis was determined, or if serum calcium levels remained under 9 mg/dL after 6 months of Dicamax D or CAL-D3 administration.

Osteoporosis drugs were administered if the patient agreed after diagnosis of osteoporosis.

This study was approved by the ethics committee of the institutional review board (approval number: 10-2018-62-071).

### Data collection

We collected a variety of data, including age at gastrectomy, sex, height, body weight, preoperative blood chemistry tests, and several records encompassing the type of operation (distal gastrectomy [DG], proximal gastrectomy [PG], pylorus-preserving gastrectomy [PPG], or total gastrectomy [TG]), tumor stage, the presence of hypertension and diabetes mellitus, and whether adjuvant chemotherapy was administered. Postoperative complications were classified using the Clavien-Dindo classification (grade ≥ 2) [25].

Postoperative records included body weight, body mass index (BMI), and blood chemistry tests measured for up to 12 months.

Blood chemistry tests from peripheral blood samples were acquired and assessed for serum albumin (Alb), cholesterol, blood urea nitrogen (BUN), creatinine (Cr), hemoglobin (Hb), calcium (Ca), phosphorous (P), alkaline phosphatase (ALP) and vitamin D.

The socioeconomic status was categorized into two groups based on the medical security type: the high-income group which is composed of patients covered by the insurance system that belonged to the National Health Service, and the low-income group which consists of those covered by the medical benefits system [26].

### Bone mineral density (BMD) measurement

Bone mineral density (BMD) was measured at the lumbar spine, femoral neck, and total hip using dual-energy X-ray absorptiometry (DXA) (GE Prodigy; Lunar Radiation, Madison, WI, USA). The T-score (g/cm²) was defined as the absolute value relative to that of young adults. Osteoporosis was defined as a T-score less than − 2.5 standard deviations (SD), and osteopenia was diagnosed if the T-score fell within the range of -1.0 to -2.5 SD, based on the criteria of the World Health Organization.

Patients were advised to undergo DXA once a year, and the procedure was conducted if they consented. DXA scans were generally initiated starting 1 year postoperatively. However, if there were results from scans performed within the first postoperative year due to necessity by other departments or as part of a health check-up, those results were also included in the analysis. The minimum imaging interval was 2 years for patients with normal BMD and 1 year for those diagnosed with osteoporosis or osteopenia. All DXA scans obtained up to 84 months after surgery were included in the analysis.

### Statistical analysis

A comparison was performed between the group with osteoporosis and the group without osteoporosis during the follow-up period. The minimum observed T-score was also recorded and used for analysis. Osteoporosis diagnosed within 12 months after gastrectomy can be from other reasons, not only gastrectomy. Therefore, we additionally investigated risk factors for patients who got osteoporosis after 12 months after gastrectomy.

We used the Student’s t-test or Mann-Whitney U test to analyze the mean values of continuous variables between the two patient groups. Categorical variables were compared using Pearson’s chi-square test or Fisher’s exact test.

Multivariate logistic regression was exploited to evaluate the risk factors associated with osteoporosis after gastrectomy.

All statistical tests were two-sided unless otherwise indicated, and a significance level of *P*-value < 0.05 was adopted. All analyses were performed using SPSS version 29.0 (IBM, Armonk, New York, USA) and statistical power was calculated with R environment (version 4.4.1, Vienna, Austria).

## Results

### Osteoporosis rates and T score obtained from DXA

**Fig. 1:**
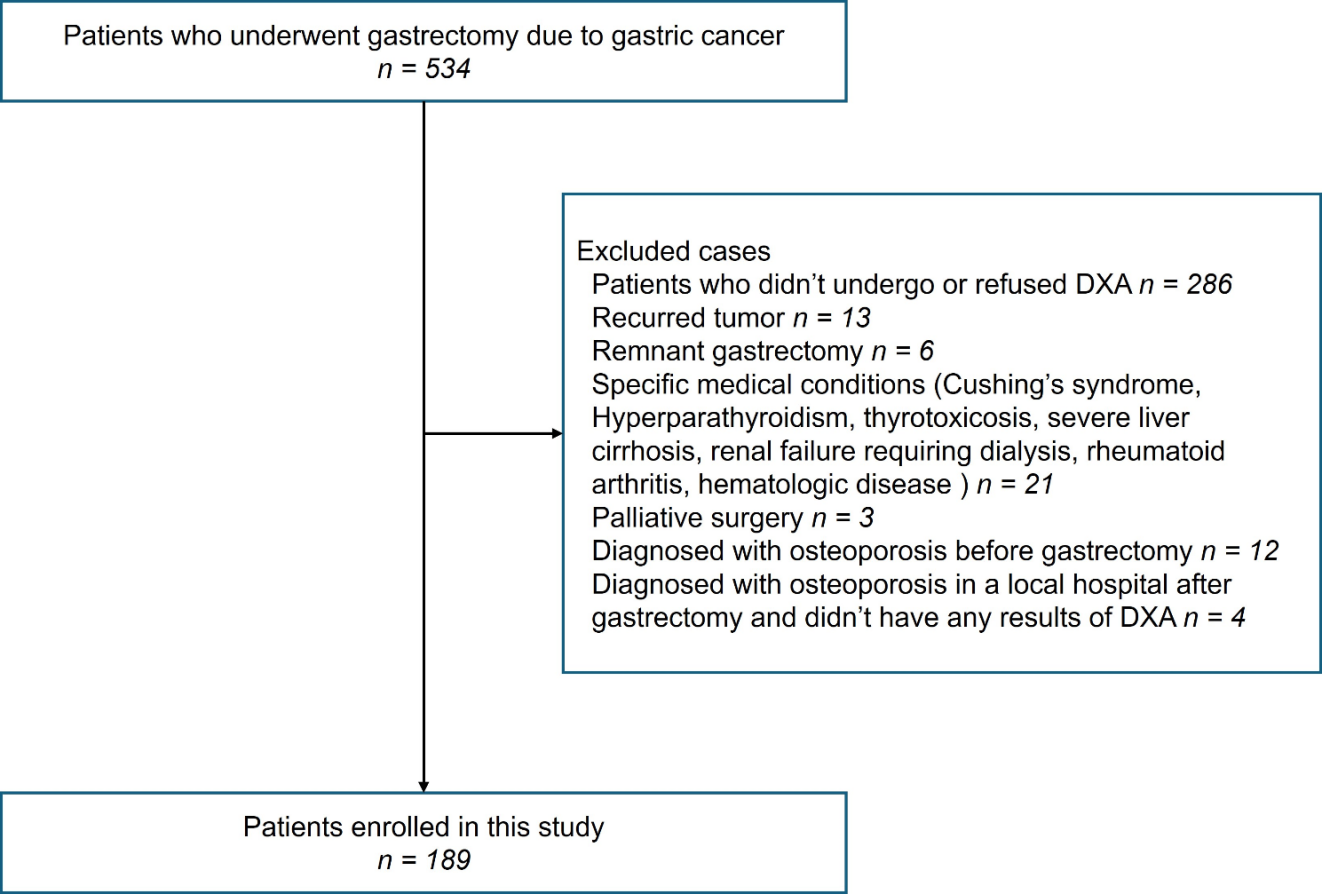
Flow chart of patient enrollment

In total, 189 patients were included in the analyses after patient enrollment. (Fig. 1) Table 1 presents the clinical characteristics of the study population who underwent gastrectomy for gastric cancer.

**Table 1:** Clinical characteristics of the study population (*n* = 189)

Variable	Mean (SD) or *N* (%)	
Age (years)	65.5	(10.3)
**SEX**		
male	125	(66.1)
female	64	(33.9)
Preoperative weight (kg)	63.1	(10.6)
Preoperative BMI (kg/m²)	24.2	(3.5)
**Socioeconomic status**		
high-income group	170	(89.9)
low-income group	19	(10.1)
**HTN**		
No	97	(51.3)
Yes	92	(48.7)
**DM**		
No	146	(77.2)
Yes	43	(22.8)
**Preop Lab**		
Hb (g/dL)	13.2	(1.9)
Alb (g/dL)	4.1	(0.3)
Cholesterol (mg/dL)	175.2	(35.7)
BUN (mg/dL)	14.1	(5.0)
Cr (mg/dL)	0.82	(0.22)
Ca (mg/ dL)	8.9	(0.4)
P (mg/dL)	3.5	(0.6)
ALP (IU/L)	73.9	(20.8)
vit D (IU)	18.1	(12.5)
**Type of operation**		
DG	135	(71.4)
TG	20	(10.6)
PG	11	(5.8)
PPG	23	(12.2)
**Postop CTx**		
No	149	(78.8)
Yes	40	(21.2)
**Stage**		
l	140	(74.1)
ll	29	(15.3)
ll	20	(10.6)
**Postop complication**		
Clavien-Dindo class < 2	166	(87.8)
Clavien-Dindo class ≥ 2	23	(12.2)

HTN: hypertension; DM: diabetes mellitus; Alb: albumin (n=185); cholesterol (n=176); BUN: blood urea nitrogen; Cr: creatinine; Hb: hemoglobin; Ca: calcium; P: phosphorous; ALP: alkaline phosphatase; vit D: vitamin D (n=26); DG: distal gastrectomy; TG: total gastrectomy; PG: proximal gastrectomy; PPG: pylorus-preserving gastrectomy; CTx: chemotherapy

A total of 72 patients (38.1%) were diagnosed with osteoporosis, while 91 patients (48.1%) were diagnosed with osteopenia. Osteoporosis was first diagnosed at an average of 29.3 months postoperatively, and 55 patients were administered osteoporosis medication, on average, 38.75 months after surgery. Calcium and vitamin D supplements were prescribed to 157 patients (83.1%) for an average of 32.4 months. Among them, 70 patients were diagnosed with osteoporosis.

**Fig. 2:**
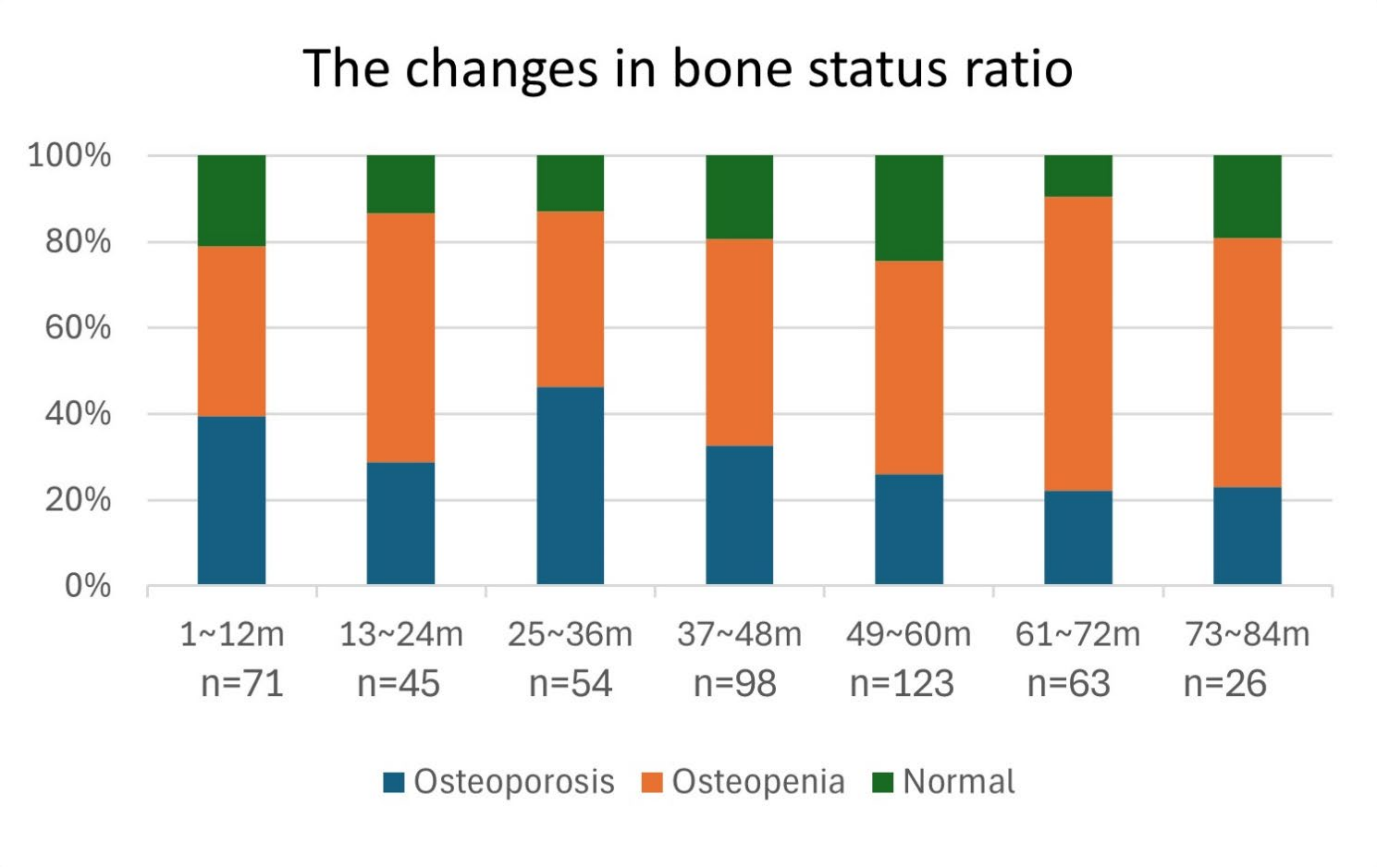
The changes in bone status ratio after gastrectomy in patients with gastric cancer. m: months

**Fig. 3:**
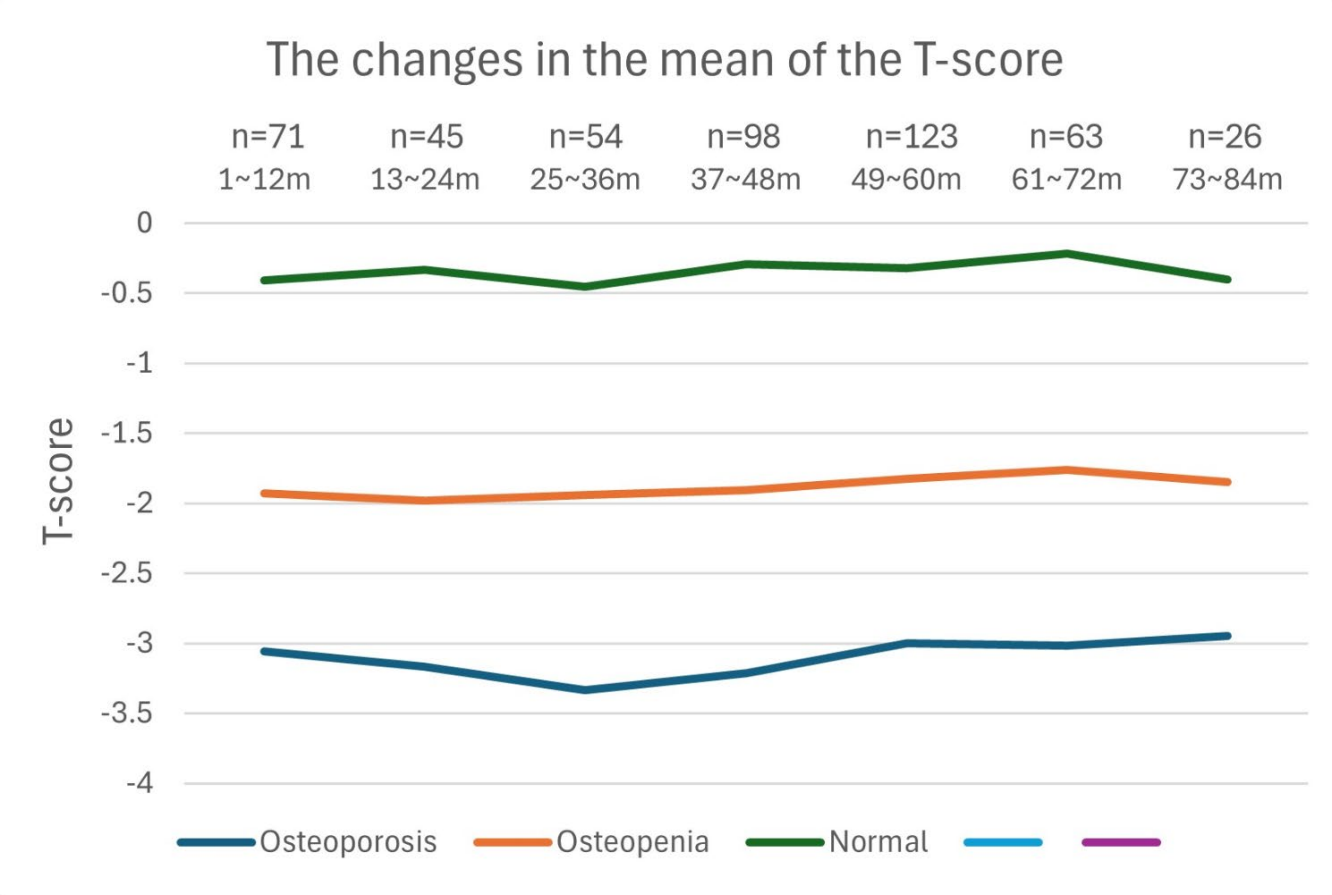
The changes in the mean of the T score after gastrectomy in patients with gastric cancer. m: months

Figure 2 indicates the proportion of patients diagnosed with osteoporosis, osteopenia, or normal over time. Figure 3 shows the average T score between those diagnosed with osteoporosis, osteopenia, or normal over time. Notably, at 36 months after follow-up, the proportion of osteoporosis patients peaked (46.3%; 24 out of 54) and the average T-score of osteoporosis patients reached its lowest value (the mean of T-score = -3.34).

### Comparison of factors associated with osteoporosis after gastrectomy

Table 2 shows the preoperative risk factors associated with osteoporosis after gastrectomy. Age (*P* < 0.001) and preoperative ALP (*P* = 0.003) were significantly higher in the osteoporosis group. Preoperative body weight was significantly lower in the osteoporosis group (*P* < 0.001). In addition, sex was associated with osteoporosis significantly (*P* < 0.001). Socioeconomic status was not associated with osteoporosis after gastrectomy.

**Table 2:** Preoperative factors associated with osteoporosis after gastrectomy

Variable	osteoporosis after gastrectomy					
	Yes	(*n* = 72)	No	(*n* = 117)	Odds Ratio	*P*-value*
	*Mean*	*(SD)*				
Age (years)	69.17	(9.0)	63.2	(10.4)		< 0.001
Preoperative weight (kg)	59.7	(9.6)	64.8	(9.7)		< 0.001
Preoperative BMI (kg/m²)	23.9	(3.8)	24.4	(3.2)		0.273
**Preop Lab**						
Hb (g/dL)	13.07	(1.95)	13.33	(1.77)		0.341
Alb (g/dL)	4.04	(0.34)	4.12	(0.29)		0.100
Cholesterol (mg/dL)	176.5	(36.2)	178.6	(35.1)		0.701
BUN (mg/dL)	13.5	(3.8)	14.6	(5.3)		0.153
Cr (mg/dL)	0.78	(0.20)	0.83	(0.22)		0.091
Ca (mg/dL)	8.86	(0.391)	8.96	(0.441)		0.110
P (mg/dL)	3.58	(0.652)	3.46	(0.553)		0.180
ALP (IU/L)	80.94	(20.34)	71.97	(20.04)		0.003
	*n*	*(%)*				
**SEX**						
male	37	(29.6)	88	(70.4)	Reference	
female	35	(54.7)	29	(45.3)	2.870 (1.538–5.359)	< 0.001
**Socioeconomic status**						
high-income group	63	(37.1)	107	(61.9)	Reference	
low-income group	9	(47.4)	10	(52.6)	1.529 (0.590–3.964)	0.380
**HTN**						
Yes	33	(35.9)	59	(64.1)	0.832 (0.462–1.498)	0.539
No	39	(40.2)	58	(59.8)	Reference	
**DM**						
Yes	15	(34.9)	28	(65.1)	0.836 (0.411–1.701)	0.622
No	57	(39.0)	89	(61.0)	Reference	
**Type of operation**						
DG	51	(37.8)	84	(62.2)	Reference	
TG	10	(50.0)	10	(50.0)	1.647 (0.641–4.229)	0.296
PG	5	(45.5)	6	(54.5)	1.373 (0.398–4.728)	0.749†
PPG	6	(26.1)	17	(73.9)	0.581 (0.215–1.570)	0.280
**Postop CTx**						
Yes	15	(37.5)	25	(62.5)	0.968 (0.471–1.990)	0.930
No	57	(38.3)	92	(61.7)	Reference	
**Stage**						
l	51	(36.4)	89	(63.6)	Reference	
ll	15	(51.7)	14	(48.3)	1.870 (0.835–4.185)	0.124
lll	6	(30.0)	14	(70.0)	0.748 (0.271–2.067)	0.574
**Postop complication**						
Clavien-Dindo class < 2	64	(38.6)	102	(61.4)	Reference	
Clavien-Dindo class ≥ 2	8	(34.8)	15	(65.2)	0.850 (0.341–2.118)	0.821

*P value for comparisons between two groups according to osteoporosis after gastrectomy

†Fisher’s exact test was used

The data enclosed between parenthesis represents the intervals of confidence at 95%. Alb: albumin; BUN: blood urea nitrogen; Cr: creatinine; Hb: hemoglobin; Ca: calcium; P: phosphorous; ALP: alkaline phosphatase; HTN: hypertension; DM: diabetes mellitus; DG: distal gastrectomy; TG: total gastrectomy; PG: proximal gastrectomy; PPG: pylorus-preserving gastrectomy; CTx: chemotherapy

Among patients diagnosed with osteoporosis, the majority (28 patients, 38.9%) were first diagnosed with osteoporosis less than 12 months after surgery. Since more than half of the patients were diagnosed with osteoporosis after 12 months, a univariate analysis of risk factors at 12 months was performed as shown in Table 3. According to Table 3, age (*P* = 0.005), body weight (*P* = 0.020), serum Ca (*P* = 0.003), Cr (*P* = 0.041), Alb (*P* = 0.016), and ALP (*P* = 0.024) at 12 months after surgery were significant.

**Table 3:** Postoperative factors associated with osteoporosis 12months after gastrectomy

Variable	osteoporosis after gastrectomy					
	Yes	(*n* = 44)*	No	(*n* = 117)	Odds Ratio	*P*-value†
	*Mean*	*(SD)*				
Age (years)	69.23	(8.9)	64.2	(10.4)		0.005
bwt#12 (kg)	56.9	(7.7)	60.3	(8.4)		0.020
BMI#12 (kg/m²)	22.2	(3.2)	22.7	(2.8)		0.309
**12months postop Lab**						
Ca#12 (mg/dL)	8.84	(0.396)	9.06	(0.413)		0.003
P#12 (mg/dL)	3.72	(0.482)	3.66	(0.576)		0.511
BUN#12 (mg/dL)	14.2	(4.3)	14.8	(4.1)		0.371
Cr#12 (mg/dL)	0.74	(0.18)	0.83	(0.22)		0.041
Alb#12 (g/dL)	4.08	(0.31)	4.21	(0.29)		0.016
Cholesterol#12 (mg/dL)	159.2	(30.3)	166.8	(35.4)		0.209
ALP#12 (IU/L)	98.95	(27.45)	87.60	(28.46)		0.024
Hb#12 (g/dL)	13.00	(1.26)	13.43	(1.50)		0.086
	*n*	*(%)*				
**SEX**						
male	27	(23.5)	88	(76.5)	Reference	
female	17	(37.0)	29	(63.0)	1.911 (0.914–3.996)	0.116
**Socioeconomic status**						
high-income group	41	(27.7)	107	(72.3)	Reference	
low-income group	3	(23.1)	10	(79.6)	0.783 (0.205–2.989)	1.000‡
**HTN**						
Yes	20	(25.3)	59	(74.7)	0.819 (0.409–1.642)	0.600
No	24	(29.3)	58	(70.7)	Reference	
**DM**						
Yes	8	(22.2)	28	(77.8)	0.706 (0.294–1.696)	0.527
No	36	(28.8)	89	(71.2)	Reference	
**Type of operation**						
DG	35	(29.4)	84	(70.6)	Reference	
TG	3	(23.1)	10	(76.9)	0.720 (0.187–2.775)	0.766‡
PG	3	(33.3)	6	(66.7)	1.200 (0.284–5.069)	1.000‡
PPG	3	(15.0)	17	(85.0)	0.424 (0.117–1.537)	0.278
**Postop CTx**						
Yes	7	(21.9)	25	(78.1)	0.680 (0.213–2.173)	0.596
No	37	(28.7)	92	(71.3)	Reference	
**Stage**						
l	34	(27.6)	89	(72.4)	Reference	
ll	7	(33.3)	14	(66.7)	1.309 (0.487–3.521)	0.607
lll	3	(17.6)	14	(82.4)	0.561 (0.152–2.075)	0.559†
**Postop complication**						
Clavien-Dindo class < 2	40	(28.2)	102	(71.8)	Reference	
Clavien-Dindo class ≥ 2	4	(21.1)	15	(78.9)	0.680 (0.213–2173)	0.596

*The number of patients diagnosed with osteoporosis 12 months after surgery

†*P* value for comparisons between two groups according to osteoporosis after gastrectomy

‡Fisher’s exact test was used

The data enclosed between parenthesis represents the intervals of confidence at 95%. #m: at # months after gastrectomy, Alb: albumin; BUN: blood urea nitrogen; Cr: creatinine; Hb: hemoglobin; Ca: calcium; P: phosphorous; ALP: alkaline phosphatase; HTN: hypertension; DM: diabetes mellitus; DG: distal gastrectomy; TG: total gastrectomy; PG: proximal gastrectomy; PPG: pylorus-preserving gastrectomy; CTx: chemotherapy

Table 4 provides the logistic regression analysis for factors associated with osteoporosis after gastrectomy. For comparison, factors with a *P*-value ≤ 0.1 from the data obtained before and within 12 months after surgery were included in the multivariate logistic regression analysis. Before surgery, age (*P* = 0.002; Adjusted OR: 1.059, 95% CI: 1.020–1.098), body weight (*P* = 0.009; Adjusted OR: 0.950, 95% CI: 0.914–0.987), sex (*P* = 0.021; Adjusted OR: 2.322, 95% CI: 1.138–4.739), and serum ALP (*P* = 0.009; Adjusted OR: 1.023, 95% CI: 1.006–1.040) were significant. At 12 months after surgery, age (*P* = 0.005; Adjusted OR: 1.067, 95% CI: 1.020–1.116), serum Ca (*P* = 0.046; Adjusted OR: 0.357, 95% CI: 0.130–0.980), Cr (*P* = 0.003; Adjusted OR: 0.021, 95% CI: 0.002–0.268), and ALP (*P* = 0.014; Adjusted OR: 1.017, 95% CI: 1.003–1.030) were observed significantly. All results of the statistical power of the analyses are exhibited in Supplementary Table 1.

**Table 4:** Multivariate logistic regression model from Tables 2 and 3

Variables	Adjusted OR (95% CI)		*P*-value*
**Preop**			
Age (years)	1.059	(1.020–1.098)	0.002
Preoperative weight (kg)	0.950	(0.914–0.987)	0.009
**SEX**			
male	1		
female	2.322	(1.138–4.739)	0.021
**Preop Lab**			
Cr (mg/dL)	0.685	(0.100-4.693)	0.700
Alb (g/dL)	0.683	(0.209–2.238)	0.529
ALP (IU/L)	1.023	(1.006–1.040)	0.009
**Postop#12m**			
Age (years)	1.067	(1.020–1.116)	0.005
bwt#12 (kg)	0.968	(0.920–1.019)	0.213
**Postop Lab**			
Ca#12 (mg/dL)	0.357	(0.130–0.980)	0.046
Cr#12 (mg/dL)	0.021	(0.002–0.268)	0.003
Alb#12 (g/dL)	0.89	(0.150–5.268)	0.898
ALP#12 (IU/L)	1.017	(1.003–1.030)	0.014
Hb#12 (g/dL)	1.248	(0.893–1.744)	0.195

*P value for comparisons between two groups according to osteoporosis after gastrectomy

The data enclosed between parenthesis represents the intervals of confidence at 95%

#m: at # months after gastrectomy; BMI: body mass index; Alb: albumin; BUN: blood urea nitrogen; Cr: creatinine; Hb: hemoglobin; Ca: calcium; P: phosphorous; ALP: alkaline phosphatase

## Discussion

This study is a retrospective analysis of whether osteoporosis is observed after gastric cancer surgery, exploring both preoperative and postoperative (up to 12 months) risk factors. Additionally, the study examined changes in the proportion and mean T-score of osteoporosis patients up to 84 months after gastrectomy, confirming that the number of osteoporosis patients peaked at 36 months. The identified risk factors for osteoporosis after gastrectomy included advanced age, female sex, and lower body weight. Furthermore, it was confirmed that high ALP, low Cr, and low Ca before or 12 months after surgery could be risk factors.

Our study is consistent with previous research on 250 long-term gastric cancer survivors, followed for approximately 5 years after gastrectomy [11]. That study reported that the risk of osteoporosis increased with age, female sex, high ALP, low BMI, and a weight change of more than 20% after gastrectomy. In addition, a prospective study focusing on patients for 3 years after gastrectomy confirmed that age, female sex, low BMI, and a history of fractures are risk factors associated with osteoporosis, which aligns with the findings of our study [27].

In our study, the maximum proportion and lowest mean T-score of osteoporosis were observed at 36 months after surgery. Based on this finding, early prediction is necessary before the incidence of osteoporosis reaches its peak. This result is also similar to that of a prospective study on changes in bone metabolism in male patients after gastrectomy, which reported a continuous decrease in T-scores for up to 24 months [28]. However, another study on the risk prediction of osteoporosis after gastrectomy found that the frequency of osteoporosis peaked at 12 months post-surgery, with the overall rate decreasing over time [12]. This discrepancy may be attributed to confounding factors, as patients in that study were prescribed Ca and vitamin D supplements, as well as osteoporosis medications.

Body weight and BMI are among the most important factors associated with osteoporosis, and a low BMI is listed as a risk factor for primary osteoporotic fractures by the World Health Organization [29, 30]. In our study, low preoperative body weight may be a predictor of an increased likelihood of osteoporosis after gastric cancer surgery. Therefore, maintaining a proper weight before gastrectomy is crucial.

ALP has previously been studied as a marker of bone formation; however, ALP alone cannot diagnose or predict osteoporosis, as other tissues, such as the liver and intestines, also contain this enzyme [31]. Nevertheless, several studies have shown that ALP is one of the risk factors for osteoporosis after gastrectomy [10, 11]. In addition to ALP, further research is needed to determine whether bone-specific alkaline phosphatase (BAP), type 1 collagen C-terminal telopeptide (CTX-1), and type 1 collagen cross-linked N-telopeptide (NTX-1) can serve as predictive factors for osteoporosis after gastrectomy in gastric cancer patients.

When gastrectomy is performed, some or all gastric functions are lost, and the possibility of bypassing the duodenum arises. Since calcium is primarily absorbed in the duodenum, rapid food passage and duodenal bypass due to gastrectomy result in reduced calcium absorption [32]. Furthermore, decreased gastric acidity after gastrectomy may impair calcium absorption in the intestine [8]. Some studies have demonstrated that failure to pass through the duodenum leads to a significantly higher ratio of 1,25-OH vitamin D to 25-OH vitamin D and significantly lower serum 25-OH vitamin D levels in patients undergoing total gastrectomy [33, 34]. In our study, multivariate analysis at 12 months post-gastrectomy revealed that low serum calcium was a risk factor for the development of osteoporosis. However, since calcium and vitamin D supplements are routinely prescribed according to protocol, there may be confounding factors.

In our study, patients’ medical security type was utilized to determine the risk of osteoporosis, aiming to elucidate any potential relationship with socioeconomic status. As a result of the analysis, there was no significant difference between patients in the medical benefits and patients in the insurrection system (*P* = 0.038; OR: 1.529, 95% CI: 0.590–3.964). This finding may be attributed to the fact that Korea is a developed country where access to nutrition and healthcare resources is relatively easy for all citizens, regardless of their socioeconomic status.

According to Huh et al., BMD is positively correlated with serum Cr in individuals with normal renal function [35]. Since this study also excluded patients with renal dysfunction requiring dialysis, lower serum Cr levels are more likely to be associated with osteoporosis, which is characterized by low BMD. Sarcopenia has also been identified as a risk factor for osteoporosis in previous studies [36]. Because serum Cr levels, derived from skeletal muscle, remain constant when renal function is normal, it is believed that serum creatinine is related to osteoporosis.

There are currently no clear guidelines for the management of osteoporosis following gastrectomy for gastric cancer. A 2003 report from the American Gastroenterological Association, based on patients with peptic ulcer disease, recommended follow-up DXA scans in patients 10 years after gastrectomy, as well as in postmenopausal women and men over 50 years of age [7]. However, it may be difficult to apply these recommendations directly to gastric cancer patients, who are often older than peptic ulcer patients, and who may have additional factors such as adjuvant chemotherapy. Additionally, in 2021, Park et al. developed a nomogram to predict osteoporosis in gastric cancer patients after gastrectomy, recommending DXA screening for high-risk patients within 6 or 12 months after surgery to facilitate early treatment [12]. Our study provides a foundational analysis of risk factors for predicting the likelihood of osteoporosis, using postoperative follow-up data in gastric cancer patients after gastrectomy.

This study has several limitations. First, while several factors that could affect osteoporosis were considered, due to the retrospective nature of the study, not all factors—such as diet, externally prescribed supplements, and unrecorded fractures—could be accounted for. Second, the study was conducted on a relatively small number of subjects at a single institution, which may have introduced selection bias. Third, not all patients underwent DXA imaging at set intervals. Although annual imaging was recommended, follow-up depended on the patient’s consent and test results. Fourth, there is no data on DXA performed before gastrectomy, making it difficult to determine the prevalence of osteoporosis and the baseline T-score before surgery. Lastly, while the *P*-value is significant at less than 0.05, there are cases where statistical power is insufficient. To address this, additional studies involving a larger number of patients are needed, and prospective studies are preferred over retrospective ones.

## Conclusions

Osteoporosis peaked at 36 months after gastrectomy in gastric cancer patients, despite calcium and vitamin D supplements being prescribed to 157 patients (83.1%) for an average of 32.4 months postoperatively. Age, body weight, sex, serum Ca, Cr, and ALP were associated with osteoporosis during the perioperative period. Socioeconomic status was not associated with osteoporosis after gastrectomy. The results of this study may suggest the necessity for early prescription of calcium and vitamin D supplements after radical gastrectomy, though this should be confirmed by a well-designed prospective study.

## Electronic Supplementary Material

Below is the link to the electronic supplementary material.

Supplementary Material 1

## Data Availability

The datasets generated and analysed during the current study are not publicly available because the decision was made during the IRB review process not to provide raw data to third parties but are available from the corresponding author on reasonable request.

## References

[1] National Cancer Information Center. Republic of Korea. [Internet]. [cited 2024 May 9]. https://www.cancer.go.kr/lay1/S1T648C650/contents.do/

[2] Sung H, Ferlay J, Siegel RL, Laversanne M, Soerjomataram I, Jemal A, Bray F. Global Cancer Statistics 2020: GLOBOCAN Estimates of Incidence and Mortality Worldwide for 36 Cancers in 185 Countries. CA Cancer J Clin. 2021;71(3):209–49. 10.3322/caac.21660. 33538338 10.3322/caac.21660

[3] Ferlay J, Colombet M, Soerjomataram I, Parkin DM, Piñeros M, Znaor A, Bray F. Cancer statistics for the year 2020: An overview. Int J Cancer. 2021 Apr 5.10.1002/ijc.33588. Online ahead of print. 10.1002/ijc.33588 33818764

[4] Information Committee of the Korean Gastric Cancer Association. Korean Gastric Cancer Association-Led Nationwide Survey on Surgically Treated Gastric Cancers in 2019. J Gastric Cancer. 2021;21(3):221–35. 10.5230/jgc.2021.21.e27. 34691807 10.5230/jgc.2021.21.e27 PMC8505121

[5] Liedman B. Symptoms after total gastrectomy on food intake, body composition, bone metabolism, and quality of life in gastric cancer patients–is reconstruction with a reservoir worthwhile? Nutrition. 1999;15(9):677–82. 10.1016/s0899-9007(99)00123-9. 10467612 10.1016/s0899-9007(99)00123-9

[6] Jun JH, Yoo JE, Lee JA, Kim YS, Sunwoo S, Kim BS, Yook JH. Anemia after gastrectomy in long-term survivors of gastric cancer: a retrospective cohort study. Int J Surg 2016 Apr:28:162–8. 10.1016/j.ijsu.2016.02.084 10.1016/j.ijsu.2016.02.084 26931339

[7] Bernstein CN, Leslie WD, Leboff MS. AGA technical review on osteoporosis in gastrointestinal diseases. Gastroenterology. 2003;124(3):795–841. 10.1053/gast.2003.50106. 12612917 10.1053/gast.2003.50106

[8] Kopic S, Geibel JP, Gastric, Acid. Calcium Absorption, and Their Impact on Bone Health. Physiol Rev. 2013;93(1):189–268. 10.1152/physrev.00015.2012. 23303909 10.1152/physrev.00015.2012

[9] Lim JS, Kim SB, Bang HY, Cheon GJ, Lee JI. High prevalence of osteoporosis in patients with gastric adenocarcinoma following gastrectomy. World J Gastroenterol. 2007;13(48):6492–7. 10.3748/wjg.v13.i48.6492. 18161918 10.3748/wjg.v13.i48.6492 PMC4611287

[10] Namikawa T, Yokota K, Iwabu J, Munekage M, Uemura S, Tsujii S, Maeda H, Kitagawa H, Karashima T, Kumon M, Inoue K, Kobayashi M, Hanazaki K. Incidence and risk factors of osteoporotic status in outpatients who underwent gastrectomy for gastric cancer. JGH Open. 2020;4(5):903–8. 10.1002/jgh3.12347. 33102762 10.1002/jgh3.12347 PMC7578282

[11] Yoo SH, Lee JA, Kang SY, Kim YS, Sung SW, Kim BS, Yook JH. Risk of osteoporosis after gastrectomy in long-term gastric cancer survivors. Gastric Cancer. 2018;21(4):720–7. 10.1007/s10120-017-0777-7. 29164360 10.1007/s10120-017-0777-7

[12] Park KB, Jeon CH, Lee HH, Chin HM, Song KY. Prediction of risk of osteoporosis after gastrectomy for gastric cancer. BJS Open. 2021;5(6):zrab123. 10.1093/bjsopen/zrab123. 34931227 10.1093/bjsopen/zrab123 PMC8688769

[13] Du Y, Zhao LJ, Xu Q, Wu KH, Deng HW. Socioeconomic status and bone mineral density in adults by race/ethnicity and gender: the Louisiana osteoporosis study. Osteoporos Int. 2017;28(5):1699–709. 10.1007/s00198-017-3951-1. 28236128 10.1007/s00198-017-3951-1

[14] Mahmud M, Muscatello DJ, Rahman MB, Osborne NJ. Association between socioeconomic deprivation and bone health status in the UK biobank cohort participants. Osteoporos Int 2024 May 28. 10.1007/s00198-024-07115-3 10.1007/s00198-024-07115-3 PMC11364661 38806788

[15] Ohmori N, Nomura K, Ohmori K, Kato Y, Itoh T, Takano K. Osteoporosis is more prevalent in adrenal than in pituitary Cushing’s syndrome. Endocr J. 2003;50(1):1–7. 10.1507/endocrj.50.1. 12733704 10.1507/endocrj.50.1

[16] Mazzuoli GF, D’Erasmo E, Pisani D. Primary hyperparathyroidism and osteoporosis. Aging Clin Exp Res. 1998;10:225–31. 10.1007/BF03339656. 10.1007/BF03339656 9801732

[17] Bassett J, O’Shea P, Sriskantharajah S, Rabier B, Boyde A, Howell P, Weiss R, Roux J, Malaval L, Clement-Lacroix P, Samarut J, Chassande O, Williams G. Thyroid hormone excess rather than thyrotropin deficiency induces osteoporosis in hyperthyroidism. Mol Endocrinol. 2007;21(5):1095–107. 10.1210/ME.2007-0033. 17327419 10.1210/me.2007-0033

[18] Giouleme OI, Vyzantiadis TA, Nikolaidis NL, Vasiliadis TG, Papageorgiou AA, Eugenidis NP, Harsoulis FI. Pathogenesis of osteoporosis in liver cirrhosis. Hepatogastroenterology. 2006 Nov-Dec;53(72):938–43. 17153457

[19] Schipper LG, van den Fleuren HW, Meinardi JR, Veldman BA, Kramers C. Treatment of osteoporosis in renal insufficiency. Clin Rheumatol. 2015;34(8):1341–5. 10.1007/s10067-015-2883-4. 25630310 10.1007/s10067-015-2883-4

[20] Geraci A. Osteoporosis in rheumatoid arthritis. Br Med J. 2012;2(5349):70. 10.5772/28677. PMC1872221 20789902

[21] Gaudio A, Xourafa A, Rapisarda R, Zanoli L, Signorelli SS, Castellino P. Hematological Diseases and Osteoporosis. Int J Mol Sci. 2020;21(10):3538. 10.3390/ijms21103538. 32429497 10.3390/ijms21103538 PMC7279036

[22] Bagger YZ, Tankó LB, Alexandersen P, Hansen HB, Møllgaard A, Ravn P, Qvist P, Kanis JA, Christiansen C. Two to three years of hormone replacement treatment in healthy women have long-term preventive effects on bone mass and osteoporotic fractures: the PERF study. Bone. 2004;34(4):728–35. 10.1016/j.bone.2003.12.021. 15050905 10.1016/j.bone.2003.12.021

[23] Guideline Committee of the Korean Gastric Cancer Association (KGCA), Development Working Group & Review Panel. Korean Practice Guideline for Gastric Cancer 2018: an evidence-based, multi-disciplinary approach. J Gastric Cancer. Mar; 2019;19(1):1–48. 10.5230/jgc.2019.19.e8. 30944757 10.5230/jgc.2019.19.e8 PMC6441770

[24] Pasqualetti P, Di Lauro G, Festuccia V, Giandomenico G, Casale R. Prognostic value of Pugh’s modification of Child-Turcotte classification in patients with cirrhosis of the liver. Panminerva Med. 1992;34(2):65–8. 1408330

[25] Katayama H, Kurokawa Y, Nakamura K, Ito H, Kanemitsu Y, Masuda N, Tsubosa Y, Satoh T, Yokomizo A, Fukuda H, Sasako M. Extended Clavien-Dindo classification of surgical complications: Japan Clinical Oncology Group postoperative complications criteria. Surg Today. 2016;46(6):668–85. 10.1007/s00595-015-1236-x. 26289837 10.1007/s00595-015-1236-x PMC4848327

[26] National Basic Living Sharing Act. Republic of Korea. [Act No. 19646, 2023 August 16, partially revised]. [cited 2024 May 9]. https://www.law.go.kr/LSW//lsInfoP.do?lsId=001973&ancYnChk=0#0000

[27] Kawabata R, Takahashi T, Saito Y, Nakatsuka R, Imamura H, Motoori M, Makari Y, Takeno A, Kishi K, Adachi S, Miyagaki H, Kurokawa Y, Yamasaki M, Eguchi H, Doki Y. Analysis of the risk factors for osteoporosis and its prevalence after gastrectomy for gastric cancer in older patients: a prospective study. Surg Today. 2023;53(4):435–42. 10.1007/s00595-022-02581-w. 36066746 10.1007/s00595-022-02581-w

[28] Atsumi Y, Rino Y, Wada H, Kitani Y, Ozawa Y, Aoyama T, Oshima T, Yukawa N, Yoshikawa T, Masuda M. Changes in bone metabolism after gastric cancer surgery in male patients: a prospective observational study. Gastric Cancer. 2019;22(1):237–43. 10.1007/s10120-018-0835-9. 29748875 10.1007/s10120-018-0835-9

[29] Kanis JA. Assessment of osteoporosis at the primary health-care level. Technical Report. http://www.shef.ac.uk/FRAX. 2007.

[30] Villareal DT, Fontana L, Weiss EP, Racette SB, Steger-May K, Schechtman KB, Klein S, Holloszy JO. Bone mineral density response to caloric restriction–induced weight loss or exercise-induced weight loss: a randomized controlled trial. Arch Intern Med. 2006;166(22):2502–10. 10.1001/archinte.166.22.2502. 17159017 10.1001/archinte.166.22.2502

[31] Mukaiyama K, Kamimura M, Uchiyama S, Ikegami S, Nakamura Y, Kato H. Elevation of serum alkaline phosphatase (ALP) level in postmenopausal women is caused by high bone turnover. Aging Clin Exp Res. 2015;27(4):413–8. 10.1007/s40520-014-0296-x. 25534961 10.1007/s40520-014-0296-x

[32] Baek KH, Jeon HM, Lee SS, Lim DJ, Oh KW, Lee WY, Rhee EJ, Han JH, Cha BY, Lee KW, Son HY, Kang SK, Kang MI. Short-term changes in bone and mineral metabolism following gastrectomy in gastric cancer patients. Bone. 2008;42(1):61–7. 10.1016/j.bone.2007.08.027. 17942383 10.1016/j.bone.2007.08.027

[33] Rino Y, Takanashi Y, Yamamoto Y, Inagaki D, Kawamoto M, Harada H, Ashida A, Wada H, Yamada R, Oshima T, Hatori S, Imada T. Bone disorder and vitamin D after gastric cancer surgery. Hepatogastroenterology 2007 Jul-Aug;54(77):1596–600. 10.1007/s10120-007-0439-2 17708309

[34] Rino Y, Yamamoto Y, Wada N, Yukawa N, Murakami H, Tamagawa H, Yamada T, Ohshima T, Masuda M, Imada T. Changes in vitamin D after gastrectomy. Gastric Cancer. 2007;10(4):228–33. 10.1007/s10120-007-0439-2. 18095078 10.1007/s10120-007-0439-2

[35] Huh JH, Choi SI, Lim JS, Chung CH, Shin JY, Lee MY. Lower Serum Creatinine Is Associated with Low Bone Mineral Density in Subjects without Overt Nephropathy. PLoS ONE. 2015;10(7):e0133062. 10.1371/journal.pone.0133062. 26207750 10.1371/journal.pone.0133062 PMC4514793

[36] Huh JH, Song MK, Park KH, Kim KJ, Kim JE, Rhee YM, LIM SK. Gender-specific pleiotropic bone-muscle relationship in the elderly from a nationwide survey (KNHANES lV). Osteoporos Int. 2014;25(3):1053–61. 10.1007/s00198-013-2531-2. 24150214 10.1007/s00198-013-2531-2

