# Supplementary material for: Perioperative risk factors for osteoporosis after radical gastrectomy for gastric cancer
Source: BMC Surg. 2024 Dec 27;24:420. doi: 10.1186/s12893-024-02717-4 (PMC11673358; doi:10.1186/s12893-024-02717-4)
Supplement: Supplementary file 1 — Supplementary Material 1 [file 12893_2024_2717_MOESM1_ESM.docx]

| **Supplementary Table 1** Statistical power of univariate and multivariate analysis | | | | |
| --- | --- | --- | --- | --- |
| **Variable** | Table 2 SP | Table 3 SP | Table 4 Preop SP | Table 4 Postop#12m SP |
| Age (years) | 0.983 | 0.832 | 0.061 | 0.050 |
| bwt (kg) | 0.939 | 0.660 | 0.056 | 0.047 |
| BMI (kg/m²) | 0.157 | 0.155 |  |  |
| **Lab** |  |  |  |  |
| Ca (mg/dL) | 0.357 | 0.864 |  | 0.885 |
| P (mg/dL) | 0.261 | 0.097 |  |  |
| BUN (mg/dL) | 0.354 | 0.126 |  |  |
| Cr (mg/dL) | 0.352 | 0.711 | 0.250 | 0.809 |
| Alb (g/dL) | 0.390 | 0.682 | 0.229 | 0.073 |
| Cholesterol (mg/dL) | 0.068 | 0.254 |  |  |
| ALP (IU/L) | 0.839 | 0.626 | 0.044 | 0.059 |
| Hb (g/dL) | 0.153 | 0.415 |  | 0.122 |
| **SEX** |  |  |  |  |
| male | Ref | Ref | Ref |  |
| female | 0.912 | 0.406 | 0.800 |  |
| **Socioeconomic status** |  |  |  |  |
| high-income group | Ref | Ref |  |  |
| low-income group | 0.141 | 0.784 |  |  |
| **HTN** |  |  |  |  |
| Yes | 0.094 | 0.087 |  |  |
| No | Ref | Ref |  |  |
| **DM** |  |  |  |  |
| Yes | 0.078 | 0.122 |  |  |
| No | Ref | Ref |  |  |
| **Type of op** |  |  |  |  |
| DG | Ref | Ref |  |  |
| TG | 0.179 | 0.784 |  |  |
| PG | 0.071 | 0.293 |  |  |
| PPG | 0.188 | 0.257 |  |  |
| **Postop CTx** |  |  |  |  |
| Yes | 0.051 | 0.100 |  |  |
| No | Ref | Ref |  |  |
| **Stage** |  |  |  |  |
| l | Ref | Ref |  |  |
| ll | 0.331 | 0.083 |  |  |
| lll | 0.087 | 0.965 |  |  |
| **Postop complication** |  |  |  |  |
| Clavien-Dindo class <2 | Ref | Ref |  |  |
| Clavien-Dindo class ≥2 | 0.064 | 0.100 |  |  |

SP: statistical power; #m: at # months after gastrectomy; bwt: body weight; BMI; body mass index; Alb: albumin; BUN: blood urea nitrogen; Cr: creatinine; Hb: hemoglobin; Ca: calcium; P: phosphorous; ALP: alkaline phosphatase; HTN: hypertension; DM: diabetes mellitus; DG: distal gastrectomy; TG: total gastrectomy; PG: proximal gastrectomy; PPG: pylorus-preserving gastrectomy; CTx: chemotherapy.
